# Trapezial Fractures and Associated Fractures of the Ulnar Carpus: A Ring-Bone Model

**DOI:** 10.5435/JAAOSGlobal-D-22-00270

**Published:** 2023-04-04

**Authors:** Frances E. Sharpe, Stephanie W. Holzmer, Amber Leis

**Affiliations:** From the Department of Orthopedic Surgery, Southern California Permanente Medical Group, Fontana California (Dr. Sharpe); Department of Orthopedic Surgery, Keck School of Medicine at USC (Dr. Sharpe), Los Angeles, CA; the Department of Plastic and Reconstructive Surgery, Loma Linda University, Loma Linda, CA (Dr. Holzmer); and the Department of Plastic and Reconstructive Surgery, University of California Irvine, Orange, CA (Dr. Leis).

## Abstract

Fractures of the trapezium are rare; however, the incidence may be under-reported in the literature. The incidence of ulnar-sided carpal body fractures as a concomitant injury has not been reported. Our study aimed to evaluate the incidence of trapezium fractures in conjunction with ulnar-sided carpal body fractures.

**Methods:**

Over a five-year period, our electronic records were queried and charts reporting carpal bone fractures were reviewed. All cases of trapezium fracture were evaluated further and presented.

**Results:**

Eight trapezial fractures were identified, representing 8% of all carpal fractures and 26% of all nonscaphoid carpal fractures. Of the eight trapezium fractures identified, five (62.5%) were associated with Bennett fracture and four (50%) were associated with ulnar-sided carpal fractures.

**Conclusion:**

Our study demonstrates a higher incidence of trapezial fractures than previously reported. Previously unreported concomitant ulnar-sided carpal body fractures are reported at a frequency nearly equal to that of concomitant Bennett fractures in our series. We propose a mechanism of injury where the carpal canal and overlying transverse carpal ligament function as a ring-bone construct similar to the pelvis. When a trapezium fracture is identified, we recommend additional evaluation for ulnar-sided injuries of the carpus.

Fractures of the trapezium are uncommon injuries. Of the nonscaphoid carpal fractures, trapezium fractures are the third or fourth most commonly seen. Although a few international studies suggest these fractures represent 1.5% to 3% of fractures of the hand,^1-3^ there have been no recent incidence reports from North America indicating the frequency of nonscaphoid carpal fractures, particularly those of the trapezium.

Several small series and case reports cite a frequent incidence of concomitant hand fractures associated with fractures of the trapezium, in particular the presence of a Bennett fracture.^4,5,6,7,8,9,10^ Associated ulnar-sided carpal fractures, specifically of the hook of the hamate, have also been described. These have been associated with fracture avulsion of the trapezial ridge from forces applied through the transverse carpal ligament (TCL); however, the incidence of concomitant ulnar carpal body injury has not yet been reported.^11,12,13,14,15^

Based on our observation of ulnar-sided carpal body fractures in sequential patients presenting with trapezium fractures, we reviewed our patients with trapezium fractures, with specific attention to evaluation of the ulnar carpus. We reported the 5-year incidence of these fractures in a community medical center. Concomitant injuries of the hand are reported. We also reviewed previous studies describing proposed mechanisms of injury to the trapezium. Based on our findings, we proposed a possible mechanism for the combined trapezium fracture and ulnar carpal fracture pattern, which we observed in four patients in our series.

## Methods

Institutional review board approval was obtained for this study. Over a 5-year period (2004 to 2009), the electronic medical records were queried under International Classification of Diseases, Ninth Revision diagnosis codes for carpal fractures and for Current Procedural Terminology codes for open or closed reduction of carpal fractures. Patient charts were reviewed and data collected regarding which carpal bone was fractured. All identified cases of trapezium fracture were reviewed for patient age and mechanism of injury. All imaging studies were reviewed including the radiologist report and independent evaluation by the authors. The decision to obtain advanced imaging was based on the individual treating physicians' concerns or preferences and was not discoverable to the chart reviewer.

## Results

**Figure 1:**
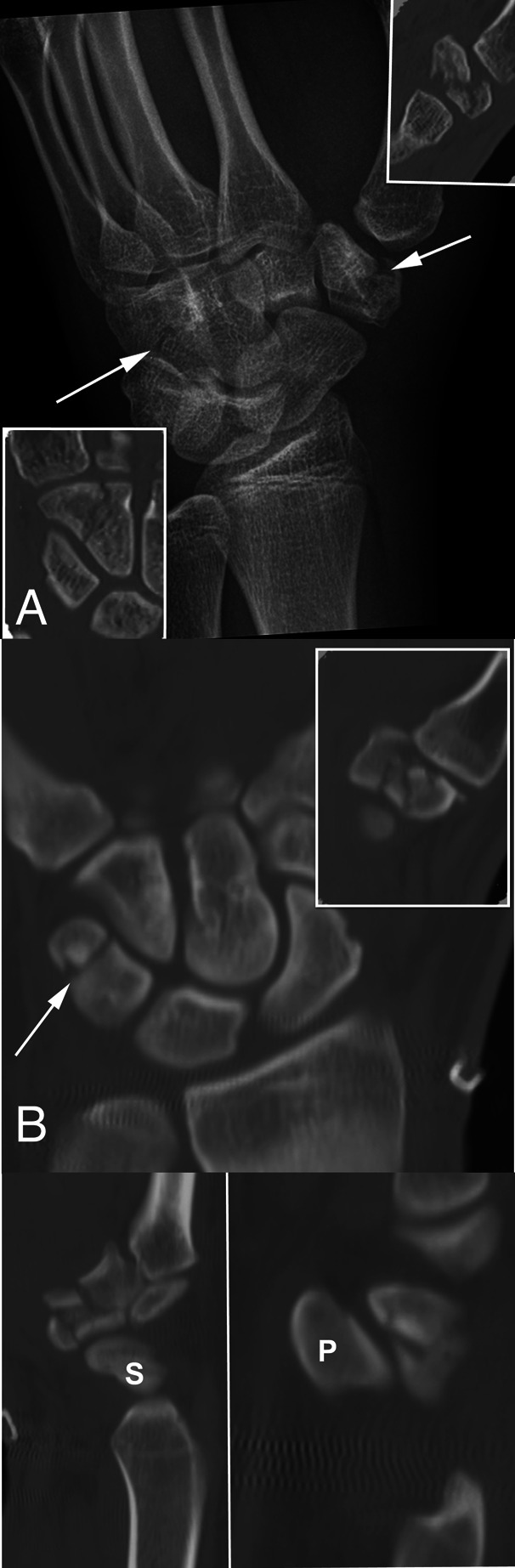
**A**, An 18-year-old right-hand–dominant man fell while riding a dirt bike. He was noted to have a comminuted fracture of the trapezium. CT was conducted to better evaluate his injury pattern. Findings included a comminuted fracture of the left trapezium with marked crush component and a nondisplaced hamate body fracture. CT images of the trapezium and hamate are shown superimposed over the planar radiograph. **B**, A 26-year-old right-hand–dominant man sustained multiple injuries from a motorcycle accident. Upper extremity injuries included bilateral open olecranon fractures, right radial head fracture, right ulnar nerve transection, and a closed right comminuted trapezium fracture with a triquetral body fracture. The trapezial body fracture was treated with open reduction and internal fixation and bone allograft augmentation. Nonsurgical care was provided for the triquetral fracture. Coronal plane CT imaging of the triquetral fracture with superimposed trapezium fracture is shown. Sagittal plane CT imaging of the trapezium (left) and triquetrum are also shown. “P” = pisiform, “S” = scaphoid

Initially, 620 patients were identified and charts were reviewed. Of these, 70 patients were subsequently excluded because they represented cases where injury was initially suspected but subsequently ruled out. Five hundred fifty-one patients remained, and all had radiographic evidence and a confirmed diagnosis of hand or wrist injury. Many cases represented noncarpal fractures and were excluded: 356 cases were metacarpal fractures, 10 were perilunate dislocations without associated fracture, 72 were fractures of the radius or ulna, and 15 were phalangeal fractures. Our chart review identified 96 cases of carpal fractures, 30 of which were nonscaphoid fractures. Of the 30 cases of nonscaphoid carpal fractures, 12 (40%) were of the hamate, 7 (23%) were of the triquetrum, 8 (27%) were of the trapezium, 2 (7%) were of the capitate, and 1 (3%) was of the pisiform. No cases of lunate or trapezoid fracture were identified. Of the eight trapezium fractures identified, one (12.5%) occurred in isolation, five (62%) were associated with a Bennett fracture, and four (50%) were associated with ulnar-sided carpal fractures. Three of these associated ulnar carpal fractures were triquetral fractures, and one was a hamate body fracture. Representative injuries are shown in Figure 1. Among all carpal fractures, including scaphoid (96 in total), trapezial fractures represented 8%, triquetrum fractures 7%, hamate fractures 12%, capitate fractures 2%, and pisiform fractures 1% of the total (Tables 1 and 2).

**Table 1:** Carpal Fracture Distribution

Carpal Bone Fractured (n = 96)	Percentage of All Carpal Fractures (%)	Percentage of Nonscaphoid Carpal Fractures (%)
Scaphoid (n = 66)	69	NA
Hamate (n = 12)	12.5	40
Trapezium (n = 8)	8.3	27
Triquetrum (n = 7)	7.3	23
Capitate (n = 2)	2.1	7
Pisiform (n = 1)	1	3

NA = not available

**Table 2:** Trapezium Fractures as Associated Injuries

Case	Age	Mechanism	Trapezial Fracture Pattern	Bennett Fracture	Ulnar Carpal Injury	Imaging
1	21	Unknown/forceful stapling	Volar trapezial ridge adjacent to the FCR groove	None	None	CT/MRI
2	14	Skateboard	Radial distal (Walker IIa)	Yes	None	CT
3	24	Altercation	Radial side (Walker IIa)	Yes	None	Planar radiographs
4	21	Football	Vertical split (Walker IV)	Yes	None	Planar radiographs
5	33	Bicycle	Vertical split with comminution (Walker V)	Yes	Dorsal triquetral avulsion/impaction	CT
6	17	MCA	Comminuted (Walker V)	Yes	Triquetral avulsion triquetral body	CT
7	26	MCA	Comminuted (Walker V)	No	Triquetral body	CT
8	18	MCA	Comminuted (Walker V)	No	Hamate body	CT

FCR = flexor carpi radialis, MCA = motorcycle accident

## Discussion

Fracture of the trapezium was first mentioned in a monograph by Destot in 1905, who stated this fracture (and fractures of the capitate and triquetrum) as “only of slight importance because of their rarity.”^16^ Kindl^17^ was the first to report on a trapezium fracture in a skier and completed his case report with a cadaver investigation to reproduce a mechanism of injury for the observed fracture. Since that time, there have been scattered case reports of trapezium fractures, often in combination with other injuries, and two large series describing proposed mechanisms of injury and fracture characteristics with outcomes of surgical treatment.^18,19^

Our series identified eight patients over a 5-year period, representing 8% of all carpal fractures and 26% of all nonscaphoid carpal fractures. Several studies cite the incidence of trapezium fractures as 5% of all fractures of the carpal bones based on a study by Cordrey in 1960. Although Cordrey and Ferrer-Torells^5^ reported this number, they gave no citation and their study was not an incidence study. Studies that have investigated the incidence of carpal fractures report the incidence of trapezium fractures between 1.2% and 3.5% of all carpal fractures.^1-3,20,21,22,23^ Van Onselen and Hove separately reported the incidence of hand fractures presenting to their emergency departments. In their studies, fractures of the hand represented 18.7% and 17.5%, respectively, of all fractures treated over a 1-year period.^3,21^ Carpal fractures were 8.2 and 14.3% of all hand fractures, and trapezium fractures were 1.4 and 2.7% of all carpal fractures, respectively (Supplemental Tables S1, http://links.lww.com/JG9/A273, and S2, http://links.lww.com/JG9/A274). Walker et al^10^ reported on 10 trapezium fractures at their institution over a 5-year period; this number is similar to what we found in our series, but we do not know the incidence of other carpal fractures during that period. The higher incidence noted in our series may represent either a higher rate of recognition because of more frequent use of advanced imaging or a higher incidence related to changes in patient activity and exposure to high-energy mechanisms of injury.

Fractures of the trapezium and their associated injuries are difficult to diagnose because of their rarity and difficulty in radiographic visualization. As such, they may be under-reported. Important clinical features of an isolated trapezium fracture include localized tenderness at the anatomic snuffbox and trapezium, pain with pinch, and difficulty maintaining pinch because of pain. Deformity is infrequent, and wrist and thumb mobility is often normal. Planar radiographic imaging should include the dorsal oblique view described by Holly^18^ or the Robert view^24^ and a carpal tunnel view.^25^ Advanced imaging using computed tomography or MRI greatly enhances the ability to diagnose these injuries and provides additional information about concomitant injuries.^26^

In our series, we identified four patients with concomitant Bennett fractures, a pattern that has been previously recognized and described, and was in fact the first fracture pattern identified in the first case report of a trapezium fracture. We also identified four patients with fractures of the ulnar carpus. This pattern has not been previously described. All fractures were nondisplaced or minimally displaced and did not require a change in the treatment plan. This fracture pattern occurred in a nearly equal frequency as the concomitant Bennett fracture and in two patients in combination with a Bennett fracture.

**Figure 2:**
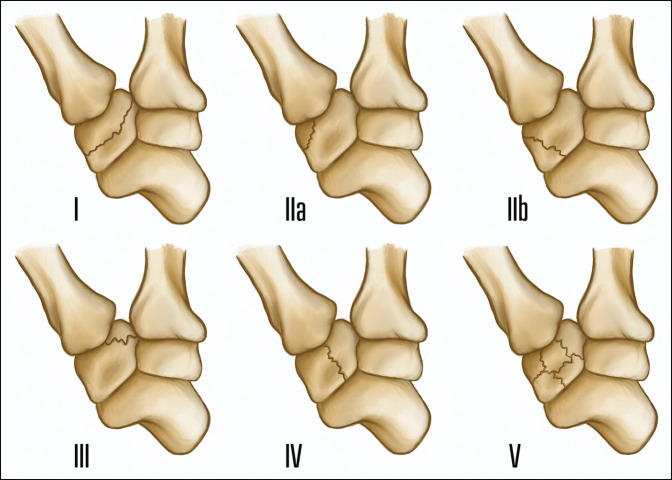
Illustration of the Walker classification system for characterization of trapezium fractures, first described in 1988.

Trapezial fractures can be classified as ridge or body fractures. Many patterns of trapezial body fractures have been described. Pointu et al reviewed the world literature on trapezium fractures, identifying 88 fractures, 34 of which had been examined by the authors. Fractures were classified as vertical, horizontal, oblique, and comminuted. Proposed mechanisms were reported as direct, indirect, and commissural shearing.^18^ Walker further classified fracture patterns based on their experience with 10 patients (Figure 2). No proposed mechanisms of body fractures were discussed in relation to the fracture pattern.^10^

**Figure 3:**
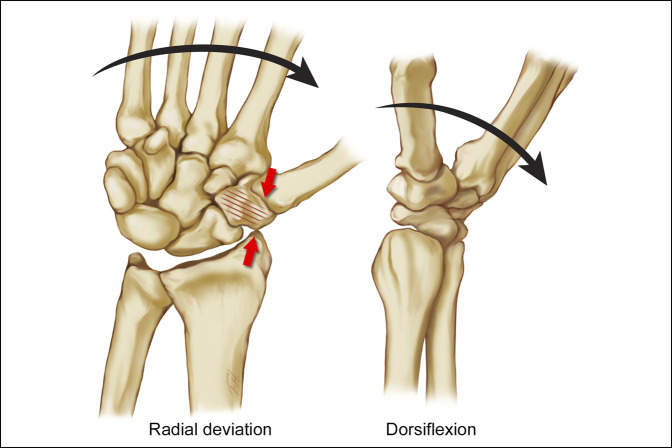
Illustration showing the “nut-cracker” mechanism of the trapezium fracture as initially described by Jeanne (1919) and Manon (1924). The trapezium is fractured by compression between the radial styloid and the first metacarpal base.

**Figure 4:**
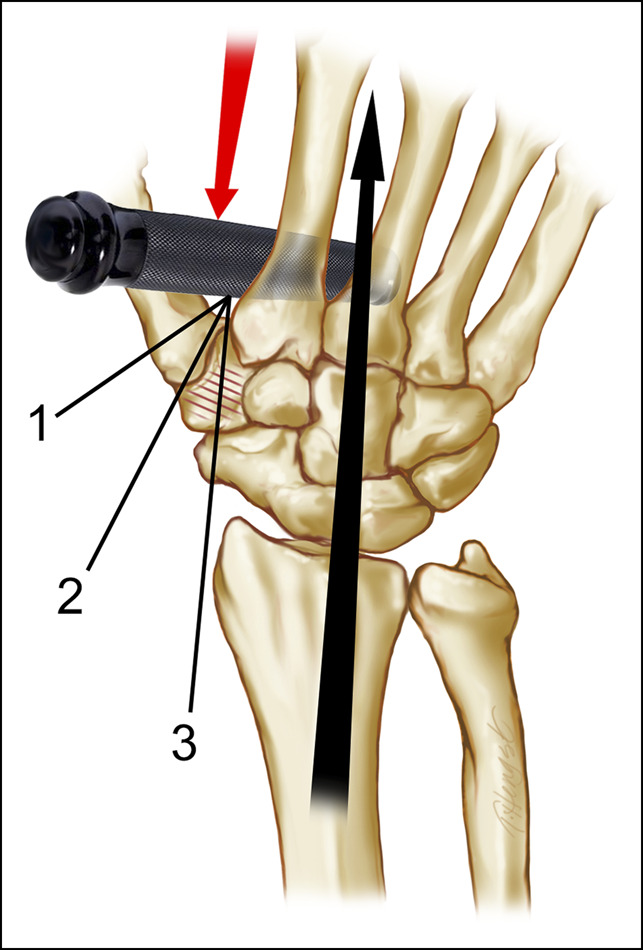
Illustration showing the first commissural shear mechanism of trapezium fracture as initially described by Monsche (1963). Load applied through the first web space may produce a Bennett fracture or trapezial fracture or both depending on the position of the thumb. Trapezial fractures from this mechanism tend to occur on the radial side (Walker IIa) or as a vertical pattern. The numerical references in the figure show the vectors of mechanical force, which would result in (1) a Bennett-type fracture, (2) a carpometacarpal dislocation, and (3) a fracture of the trapezium.

Experimental models have historically struggled to consistently re-create fractures of the trapezium. Our understanding of the mechanism of injury, therefore, comes in part from clinical cases and conjecture. Kindl first investigated the fracture of the trapezium that occurred in conjunction with a Bennett fracture in a skier. Based on this finding, he conducted a series of cadaver experiments to create a trapezial fracture in an experimental setting by applying a direct load (mallet strike) to the metacarpal while positioning it in varying degrees of abduction and adduction. Using this technique, he produced a trapezial fracture in only one of 10 specimens.^17^ Subsequent laboratory studies have also had difficulty creating a fracture by this mechanism.^10,18,27,28^ Jeanne and Manon separately showed that trapezial fractures could be produced through a “nut-cracker” mechanism, loading the wrist in dorsiflexion and radial deviation. The trapezium is compressed between the radial styloid and the base of the first metacarpal^27^ (Figure 3). A different mechanism was proposed with load occurring through the first web space, described as a commissural shearing force as might occur with gripping a steering wheel, handlebar, or ski pole (Figure 4). This position of loading can also produce a Bennett fracture, depending on the angle of the metacarpal.^10,18,28,29^

**Figure 5:**
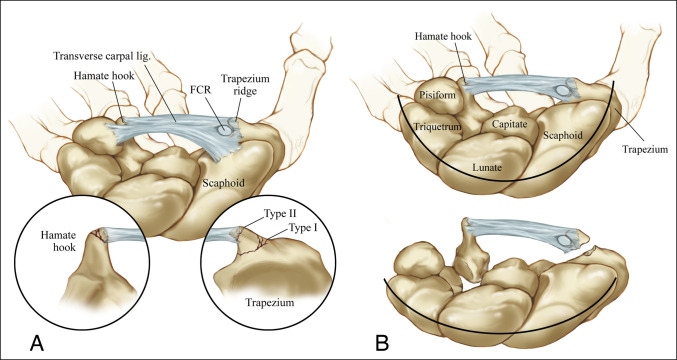
Illustration showing that load applied to the TCL can cause a spectrum of injury. **A**, Fractures of the trapezial ridge can occur with avulsion of the TCL or with a fracture of the hook of the hamate as described by Palmar (1981). Palmar proposed a classification of the trapezial ridge fracture into type I (occurring at the base of the trapezial ridge that has a higher healing capacity) and type II (occurring at the apex of the trapezial ridge and having a lower healing potential). **B**, Oshiho described a dislocation of the hamate occurring with a trapezial ridge fracture, positing a load across the TCL and flattening the transverse carpal arch. FCR = flexor carpi radialis, TCL = transverse carpal ligament

Trapezial ridge fractures have been reported to occur through loading of the TCL and often occur in conjunction with a fracture of the hook of the hamate or an avulsion of the TCL from its ulnar insertion^11,12,15^ (Figure 5). Along this spectrum of injury, Ohshio described a case of dislocation of the hamate in conjunction with a trapezial ridge fracture. The mechanism was again proposed to occur through loading of the TCL causing an avulsion fracture on the radial side and carpal dislocation on the ulnar side^14^ (Figure 5). Only one other hamate fracture was described in conjunction with trapezium fractures. The location of this fracture was not specified.^19^ In our study, treatment was not altered on discovery of concomitant ulnar carpal injuries; however, the case presented by Ohshio et al^14^ required surgical intervention for treatment of the dislocated hamate with open reduction and Kirschner wire fixation. In this particular case, advanced imaging with computed tomography was used to better characterize the injury pattern. When a trapezium fracture is identified, we recommend additional evaluation for ulnar-sided injuries of the carpus with clinical examination. CT scans are frequently used to further characterize trapezial fractures even in the absence of concomitant injury. Awareness of the possibility of ulnar-sided injuries may help guide the decision to obtain advanced imaging.

**Figure 6:**
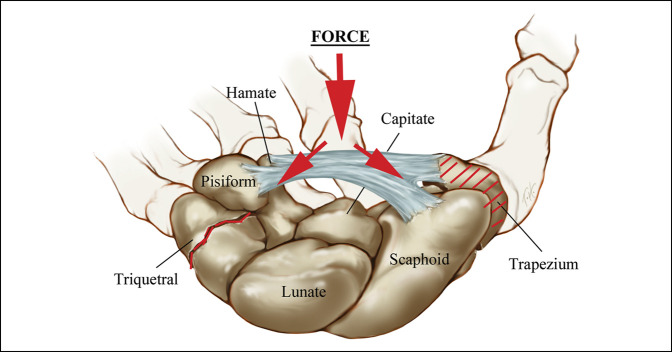
Within the spectrum of injury of load applied to the TCL, we propose a mechanism of load applied to the ring-bone construct of the carpal canal and TCL resulting in trapezial body fracture and fracture of the ulnar carpus (body fractures) of the hamate or triquetrum. TCL = transverse carpal ligament

Similar to the mechanism proposed for trapezial ridge fractures, we hypothesize that the carpal canal, along with the TCL create a ring-bone construct similar to the pelvis, such that when the ring is disrupted in one location, that there is force transmission and potential disruption to another point on the ring, thus explaining the findings in our study of a high number of ulnar carpal injuries (Figure 6). This ring-bone concept is dependent on the mechanism of loading. For example, with pure loading along the first web space, we would not expect the trapezial injury to disrupt the ring as when loading across the TCL or carpal canal. Most high-energy injuries have some component of mixed loading, which may combine both indirect and commissural loading forces. It is interesting to note that in all cases of trapezial fractures occurring in conjunction with ulnar carpal fractures in our study, a handlebar was involved or may have been involved.

The limitations of this study include our reliance on injury coding to discover all carpal bone fractures. This is likely the reason for the low number of triquetral fractures identified in this study, where a small dorsal fracture of the triquetrum might not be coded as a carpal fracture. This would in turn increase the relative incidence of trapezial fractures in our study. Additional limitations include lack of advanced imaging on all the patients; where it is possible, additional ulnar-sided carpal injuries may have been present.

Although trapezium fractures are uncommon injuries, the first carpometacarpal joint is an integral component of the radial column of the hand, making recognition and treatment of these injuries important to maintain mobility and function of the thumb. Our study demonstrates a higher incidence of these injuries than reported in the literature, likely because of increased recognition of trapezium fractures through advanced imaging. Previously unreported concomitant ulnar-sided carpal body fractures are reported at a frequency nearly equal to that of concomitant Bennett fractures in our series. We propose a mechanism of injury where the carpal canal and overlying TCL function as a ring-bone construct similar to the pelvis. Loading of this ring, usually through a direct blow to the TCL, produces disruption of the ring in two locations. This proposed mechanism may help guide future biomechanical studies, which try to simulate a trapezial fracture model.
